# Regulation of autophagy by microRNAs in human breast cancer

**DOI:** 10.1186/s12929-021-00715-9

**Published:** 2021-03-25

**Authors:** Zhi Xiong Chong, Swee Keong Yeap, Wan Yong Ho

**Affiliations:** 1 Faculty of Science and Engineering, University of Nottingham Malaysia, 43500 Semenyih, Selangor Malaysia; 2 China-ASEAN College of Marine Sciences, Xiamen University Malaysia, 43900 Sepang, Selangor Malaysia

**Keywords:** Breast cancer, miRNAs, Autophagy, Biomarkers, Therapeutic agents

## Abstract

Breast cancer is the most common solid cancer that affects female population globally. MicroRNAs (miRNAs) are short non-coding RNAs that can regulate post-transcriptional modification of multiple downstream genes. Autophagy is a conserved cellular catabolic activity that aims to provide nutrients and degrade un-usable macromolecules in mammalian cells. A number of in vitro, in vivo and clinical studies have reported that some miRNAs could modulate autophagy activity in human breast cancer cells, and these would influence human breast cancer progression and treatment response. Therefore, this review was aimed to discuss the roles of autophagy-regulating miRNAs in influencing breast cancer development and treatment response. The review would first introduce autophagy types and process, followed by the discussion of the roles of different miRNAs in modulating autophagy in human breast cancer, and to explore how would this miRNA-autophagy regulatory process affect the disease progression or treatment response. Lastly, the potential applications and challenges of utilizing autophagy-regulating miRNAs as breast cancer biomarkers and novel therapeutic agents would be discussed.

## Introduction

Breast cancer is currently the number one cancer that affects the female population worldwide and every year, more than 2 million females will be diagnosed to have this malignancy [1]. It is estimated that more than 40,000 breast cancer patients passed away because of this malignancy in year 2019 alone [2]. The challenge in managing this heterogenous malignancy is that this cancer is highly aggressive [3], and it is always associated with problems like chemoresistance [4], radioresistance [5], resistance towards hormonal therapy [6] and resistance towards targeted therapy [7].

MicroRNAs (miRNAs) are short endogenous, single-stranded, non-coding RNAs (ncRNAs) which contain 18–25 nucleotides [8, 9]. MiRNAs have been reported to play essential roles in regulating the post-transcriptional modification of multiple downstream targets [10]. By binding to the complementary sequences at the 3′-untranslated regions (3′-UTR) of the mRNAs of the target genes, miRNAs could repress the translation of these target genes [11]. With the advancement in the field of molecular biology and clinical science, in the past 20 years, many in vitro, in vivo and clinical studies have been conducted to investigate the potential roles of miRNAs as biomarkers in diagnosing and predicting the prognosis of human breast cancer [12, 13], and, as novel therapeutic agents to tackle breast cancer [14, 15].

Autophagy is a conserved, ubiquitous and important cellular degradative and catabolic activity that aims to maintain cellular homeostasis [16, 17]. This cellular process has been described for more than 30 years ago and it started to gain enormous attention worldwide when Professor Yoshinori Ohsumi was awarded with the Nobel Prize in Medicine and Physiology in year 2016 because of his distinctive works in autophagy [18]. Dysregulation of autophagy has been reported to associate with a number of communicable [19] and non-communicable diseases [20, 21]. Autophagy-related non-communicable diseases can be further divided into cancerous or non-cancerous diseases like cardiovascular disease [22], neurodegenerative disease [23, 24] and skin disease [25]. Malignancies which are related to autophagy dysregulation include colorectal cancer [21], gastric cancer [26], breast cancer [27, 28], lung cancer [29], leukemia and lymphoma [30], ovarian cancer [31], and few other cancers [32, 33].

Autophagy is essential to provide nutrients to the cancer cells to grow and at the same time, eliminate un-usable cellular macromolecules that could potentially pose harm to the cancer cells [34]. From some published in vitro, in vivo and clinical studies’ findings [35–37], regulation of autophagy by miRNAs have been demonstrated to exert some effects in influencing the human breast cancer development and treatment response. In other word, a number of miRNAs was shown to be able to either up- or down-regulate cellular autophagy, and this would eventually enhance or suppress breast cancer progression. This review, therefore, was aimed to summarize the published findings from various studies on the potential roles of miRNAs in regulating autophagy in human breast cancer, and subsequently, to discuss how does this miRNA-autophagy modulation process would influence breast cancer development and treatment response. The review would first introduce autophagy types and process, followed by the discussion on the roles of autophagy-regulating miRNAs in influencing human breast cancer development and treatment response. Lastly, the applications and challenges of employing autophagy-regulating miRNAs as potential breast cancer biomarkers and therapeutic agents would be discussed.

## Autophagy types and process, and functional roles of autophagy in breast cancer

The main objective of autophagy or “self-eating” (Greek meaning) is to break down cytoplasmic components like macromolecules and organelles, in order to sustain cellular metabolism and to ensure cellular homeostasis [16, 38, 39]. Some people described autophagy as a cellular “re-cycling process” that digests old and unwanted substances and turns it into useful nutrients for cellular usage [25]. Autophagy is important to avoid the accumulation of harmful substances like precipitated proteins, damaged cellular organelles and oncogenic materials that could pose danger to the cells [38, 39]. The failures to eliminate these unnecessary, aged or toxic substances would trigger intracellular inflammation which would generate reactive oxygen species (ROS) and subsequently, these cellular processes would lead to the development of cellular degeneration, apoptosis and carcinogenesis [38–40].

### Types of autophagy

**Fig. 1:**
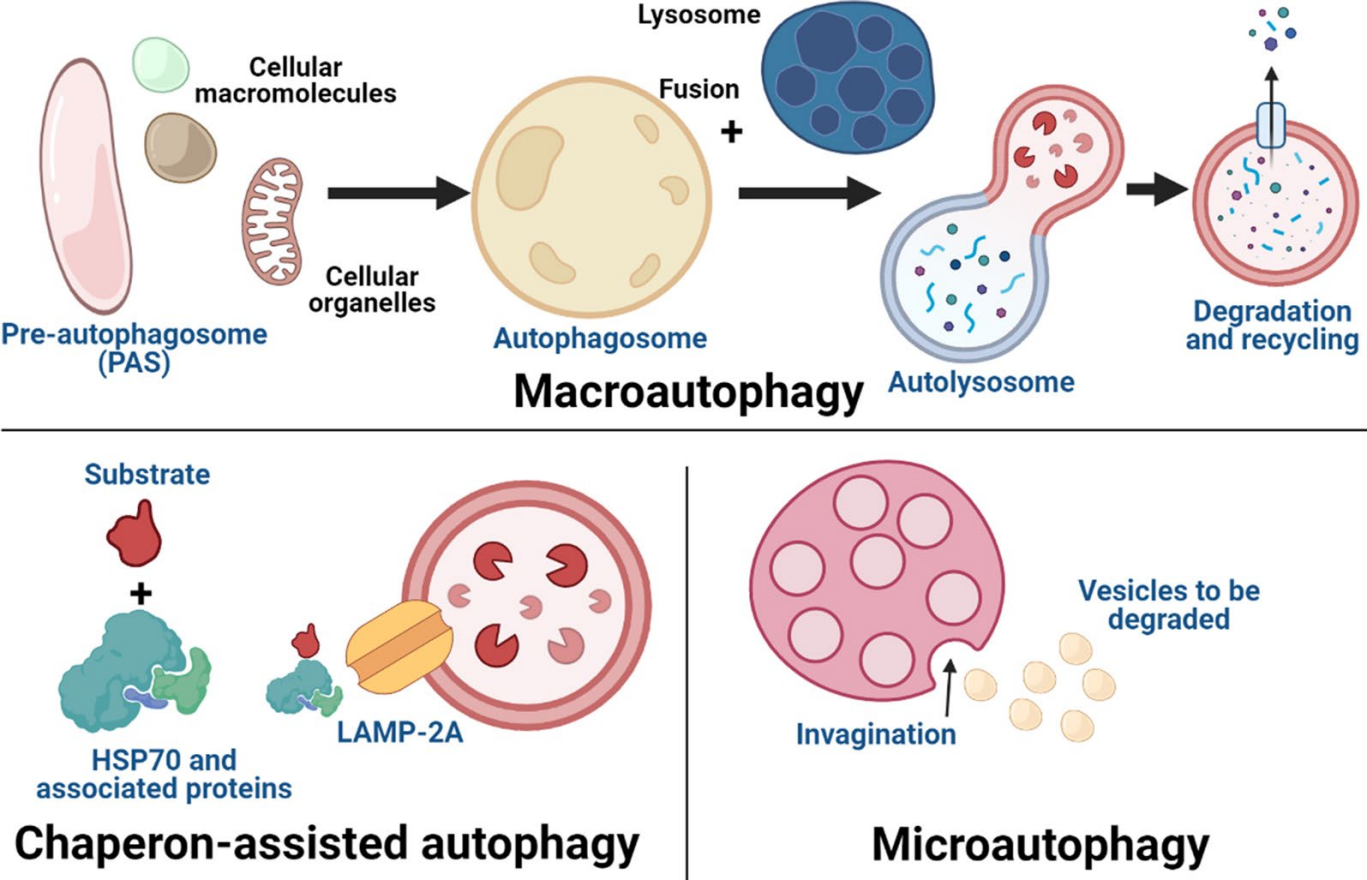
Types of autophagy. Autophagy can be divided into macroautophagy, microautophagy and chaperon-mediated autophagy (CMA) [16]. Macroautophagy is a highly conserved cellular process that involves the formation of autophagosome via sequestration of a portion of a cellular component to form autophagosome [39]. Fusion of autophagosome and lysosome forms autolysosome in which degradation of macromolecules and dysfunctional cellular organelles would take place [16]. Microautophagy involves the engulfment of a vesicle by lysosome via cytoplasmic invagination [42]. CMA is a type of highly selective autophagy in which only specific macromolecules that are tagged with a special C-terminal KFERQ motif will be recognized by specific chaperon protein (HSC70), this will then facilitate the binding of the selected macromolecules to the LAMP-A2 protein on the lysosome surface for autophagy to happen [43]

Autophagy can be generally divided into three types (Fig. 1), namely, macroautophagy, microautophagy and chaperone-mediated autophagy (CMA) [16]. Macroautophagy is an evolutionarily, highly conserved and common type of autophagy that involves the sequestration of a portion of a cellular organelle to form autophagosome [41]. Lysosome then fuses with the autophagosome to form autolysosome in which the macromolecules like proteins and organelles to be degraded will be digested within the autolysosome [39]. Selective macroautophagy is a specific type of autophagy in which selected dysfunctional cellular organelles or substrates will be recognized and selected for autophagy [38]. Examples of selective macroautophagy include mitophagy (mitochondrion), lysophagy (lysosome), pexophagy (peroxisome), ribophagy (ribosome), reticulophagy (endoplasmic reticulum) and nucleophagy (nuclear components) [38]. Microautophagy is a type of autophagy in which multiple, small vesicles are engulfed by the lysosomes via lysosomal cytoplasmic invagination [16]. The detail mechanism of microautophagy in lysosome is still unclear [42] but for endosomal microautophagy, several proteins like endosomal sorting complexes required for transport I and III (ESCRT/III) and heat shock cognate 71 kDa protein (HSC70), have been reported to play essential roles in initiating the electrostatic interaction between the protein substrates and endosomes for autophagy to happen [16]. CMA, on the other hand, is a highly selective process in which the cytoplasmic constituents that are tagged with a special C-terminal KFERQ motif will be recognized by chaperone protein like HSC70, which will then guide them to the lysosome by binding the motif to the lysosome-associated membrane protein type 2a (LAMP-A2) protein on the lysosome surface [43]. LAMP-2A is stabilized by two proteins, namely, GFAP and HSP90 [42]. LAMP-2A monomeric protein must form a multimeric complex to transport the substrate into the lysosome for degradation and this is a dynamic process which involves the monomers assembly for substrate translocation and upon completing the mission, the multimeric complex will be dissociated and degraded [16].

### Autophagy steps

**Fig. 2:**
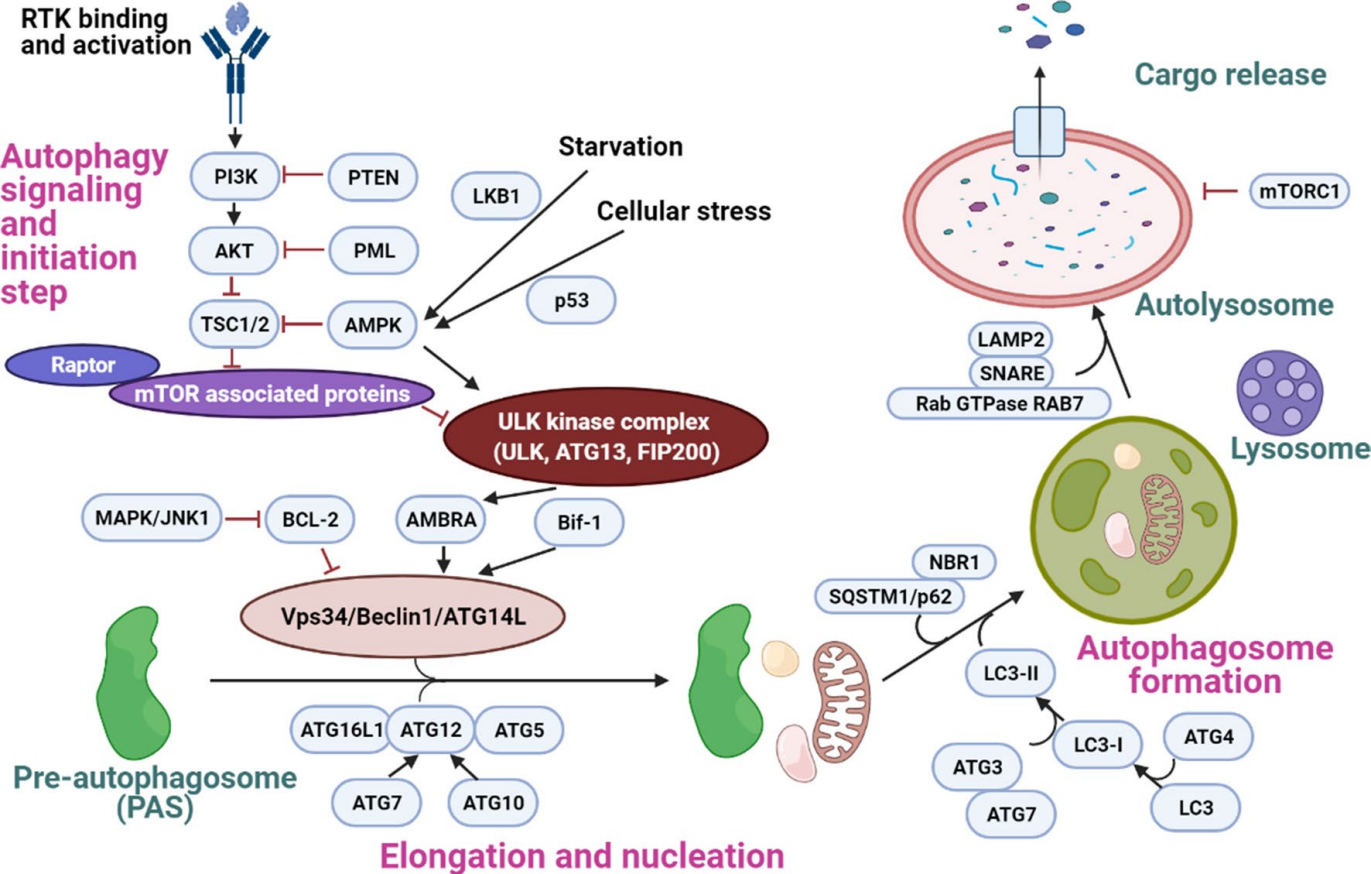
Molecular mechanism of autophagy. There are generally five steps of autophagy which include initiation, elongation, nucleation, fusion and degradation [39]. Energy deprivation or cellular stress will generate cellular autophagy signals that activate the ULK1 kinase complex to activate the downstream AMBRA protein [16]. AMBRA then phosphorylates the class III PI3K to increase PIP3 level and this helps recruiting a number of autophagy related proteins like WIPI2, ATG16L1, ATG12, ATG5 and SQSTM1/p62 to the pre-autophagosome (PAS) [16]. As these essential proteins are being recruited, PAS elongates and the production of lipidated LC3-II helps the PAS to surround and enclose the substrate macromolecules to be digested [46]. When the nucleation process is completed, mature autophagosome is formed and it is ready to bind to the lysosome to form autolysosome [39]. Positive regulators of autophagy process include AMPK [54], PTEN [56], Bif-1 [58] and TFEB [59] while negative regulators of autophagy include Akt [56], mTORC1 [60] and Bcl-2 [57]

Next, the molecular mechanism of general autophagy process (Fig. 2) will be discussed. Cellular conditions like nutrients deprivation, presence of oxidative stress or growth hormones and accumulation of senescence organelles will generate autophagy signals to the affected cells [16, 44]. The autophagy process can be generally divided into five stages including initiation, elongation, nucleation, fusion and degradation [39]. This cellular process will begin at the endoplasmic reticulum (ER) in which part of the double layer membrane of the ER will be bud off to form a cup shape substance called pre-autophagosome (PAS) [45]. A protein complex consists of Unc-51 like autophagy activating kinase (ULK1), autophagy related proteins (Atg13/Atg101) and FAK family kinase-interacting protein of 200 kDa (FIP200) will be recruited and bound to the PAS [46]. This is known as the initiation step of autophagy [46]. The ULK kinase complex phosphorylates and activates autophagy and beclin-1 regulator (AMBRA) protein, and AMBRA then phosphorylates a class III phosphoinositide 3-kinase (PI3K) complex consisting of Beclin1, ATG14L, VPS34 and VPS15 [16]. The activated PI3K complex can now convert phosphatidylinositol-4,5-bisphosphate (PIP2) to phosphatidylinositol-3,4,5-bisphosphate (PIP3) [47]. The elevation of the surrounding PIP3 concentration attracts another two proteins called WD repeat domain phosphoinositide-interacting protein (WIPI2) and zinc-finger FYVE domain-containing protein 1 (DFCP1) to the PAS membrane [46]. WIPI2 protein has been reported to play essential roles in binding the ATG16L1 protein and this aids in attracting the ATG16L1/ATG5/ATG12 protein complex to the PAS [46]. The recruitment of ATG16L1/ATG5/ATG12 complex is important in preventing the premature fusion of the autophagosome with the lysosome [16]. Besides, other proteins which are important in cargo or macromolecules sequestration like p62, sequestosome 1 (SQSTM1) and NBR1 will also be attracted and bind to the PAS [18]. SQSTM1/p62 and NBR1 are important in regulating ubiquitylation processes by facilitating the binding of the selective ubiquitinated proteins to the PAS to be removed via autophagy activity [18]. As different proteins are being recruited and bound to the PAS, it also elongates in preparation for subsequent nucleation process [39].

Once most of the macromolecules, organelles and proteins required for degradation have been recruited, a key protein that aids in the membrane closure called LC3 will come and bind to the membrane of the pre-autophagosome [48, 49]. LC3 is derived from pro-LC3 and the cleavage by cysteine protease ATG4 will produce LC3-I [50]. ATG3/ATG7 bound complex will then come and facilitate the binding of LC-3 with phosphatidylethanolamine (PE) to form PE-conjugated LC3-II [49]. Together with other autophagy related proteins, PE-conjugated LC3-II will initiate the membrane sealing to form a mature autophagosome [39, 49]. Since LC3-II protein is a vital protein involved in the autophagosome formation and nucleation process, the detection of LC3-II has been regarded as one of the usual approaches to measure autophagy activity [39]. Once the membrane of the mature autophagosome is fully formed, LC3-II will be detached and now the autophagosome is ready for fusion with the lysosome [39]. Presence of other proteins likes soluble NSF attachment protein receptor (SNARE), lysosome associated membrane protein (LAMP2) and Rab GTPase RAB 7 on the mature autophagosome and lysosome surface will facilitate the fusion with the targeted lysosome to form autolysosome [46, 51]. A number of tethers (HOPS, ATG14L and TECPR1), motor adaptors (FYCO1, PLEKHM1/2 and RILP) will also be involved in mediating the fusion process [51]. As the fusion completes, the autophagosome contents will be released and these substances will be degraded in an acidic environment containing enzymes like cathepsin B, cathepsin L and other hydrolytic enzymes [52, 53]. The digested substances will then be released from the autolysosome via protein channels and it is now can be used for anabolism or other cellular processes [16, 52, 53]. The ultimate fate of the autolysosome, however, is still poorly understood, and it is unclear whether it will dissociate into lysosome and autophagosome after the digestion process is completed [39].

There are a number of proteins which have been proven to play important roles in regulating the cellular autophagy activities [16]. Energy deprivation would raise intracellular adenosine monophosphate-activated protein kinase (AMPK) level and AMPK could phosphorylate ULK1 to activate it while inhibiting tuberous sclerosis 1/2 protein (TSC1/2) to inactivate the mTOR signaling pathway that could block activation of the ULK kinase complex [16, 54]. Protein kinase B or Akt signaling is known to inhibit autophagy induction by activating mTOR and the presence of PTEN will inhibit the PI3K/Akt signaling pathway [55]. As a result, inactivation of the Akt signaling pathway by PTEN will promote autophagy activity [56]. Bcl-2, an important regulating protein of the apoptotic pathway can bind and inhibit Beclin1 to prevent the formation of autophagosome [57]. Endophilin (Bif-1) is one of the core proteins involved in the vesicle endocytosis and it has been reported to play a vital role in interacting with Beclin-1 to regulate autophagosome formation [58]. Besides, autophagy regulation can also take place at the last phase of autophagy, which is during the autolysosome degradation process [59]. Transcription factor EB (TFEB) and mTOR complex 1 (mTORC1) are two essential proteins in coordinating the lysosome nutrient sensing machinery (LYNUS) of the lysosome [60]. Presence of abundant amino acids inside the lysosome will be sensed by vATPase, a hydrogen pump at the lysosomal membrane surface; and vATPase will relay the information to the rags proteins of the LYNUS complex [60]. When this occurs, mTORC1, which now binds to the LYNUS, will phosphorylate TFEB to inactivate it and prevent it from translocating into the nucleus [60]. Inactivation of TFEB will results in the reduction of the transcription of key genes involved in regulating endocytosis and autophagy, and downregulate the lysosomal biogenesis [59, 60]. This prevents the formation of new autolysosome via fusion of lysosome and autophagosome [60].

### Functional roles of autophagy in breast cancer

Like in other human cancer, autophagy could help in either promoting or inhibiting breast cancer [61]. A previous review has reported that autophagy plays a dynamic role in the breast cancer development, and it can be tumour-inhibiting in the early phase of cancer but become tumour-promoting in the later phase of cancer [62]. For tumour-promoting role, autophagy is an important cellular process which helps to provide nutrients and remove harmful cellular macromolecules from the breast cancer cells [63]. Thus, autophagy is said to be playing a vital role in maintaining breast cancer cells homeostasis [63], which in turn this could help the cancer cells to survive in stressful conditions like after radiotherapy or targeted therapy have been administered [63, 64]. However, autophagy can be detrimental to the breast cancer cells if it occurs excessively as autophagy is a catabolic cellular process that can lead to cellular death [65]. In some study, it has been reported that autophagy induction can be employed as one of the strategies to accelerate breast cancer cells death and to sensitize the breast cancer cells towards breast cancer therapies like chemotherapy and hormonal therapy [66]. Since autophagy is a complicated cellular process, which its role as a “friend” or “foe” in breast cancer progression is still debatable [65], more functional study is therefore needed to be conducted in the future to determine whether this cellular activity is more likely to promote or inhibit breast cancer development. Clarification of the exact role of autophagy in breast cancer development can help to strategize beneficial therapeutic options that either induce or inhibit autophagy in breast cancer patients to better eradicate this malignancy from the patients’ body.

## Autophagy regulation by miRNAs in human breast cancer: how does this influence disease progression or treatment response

**Fig. 3:**
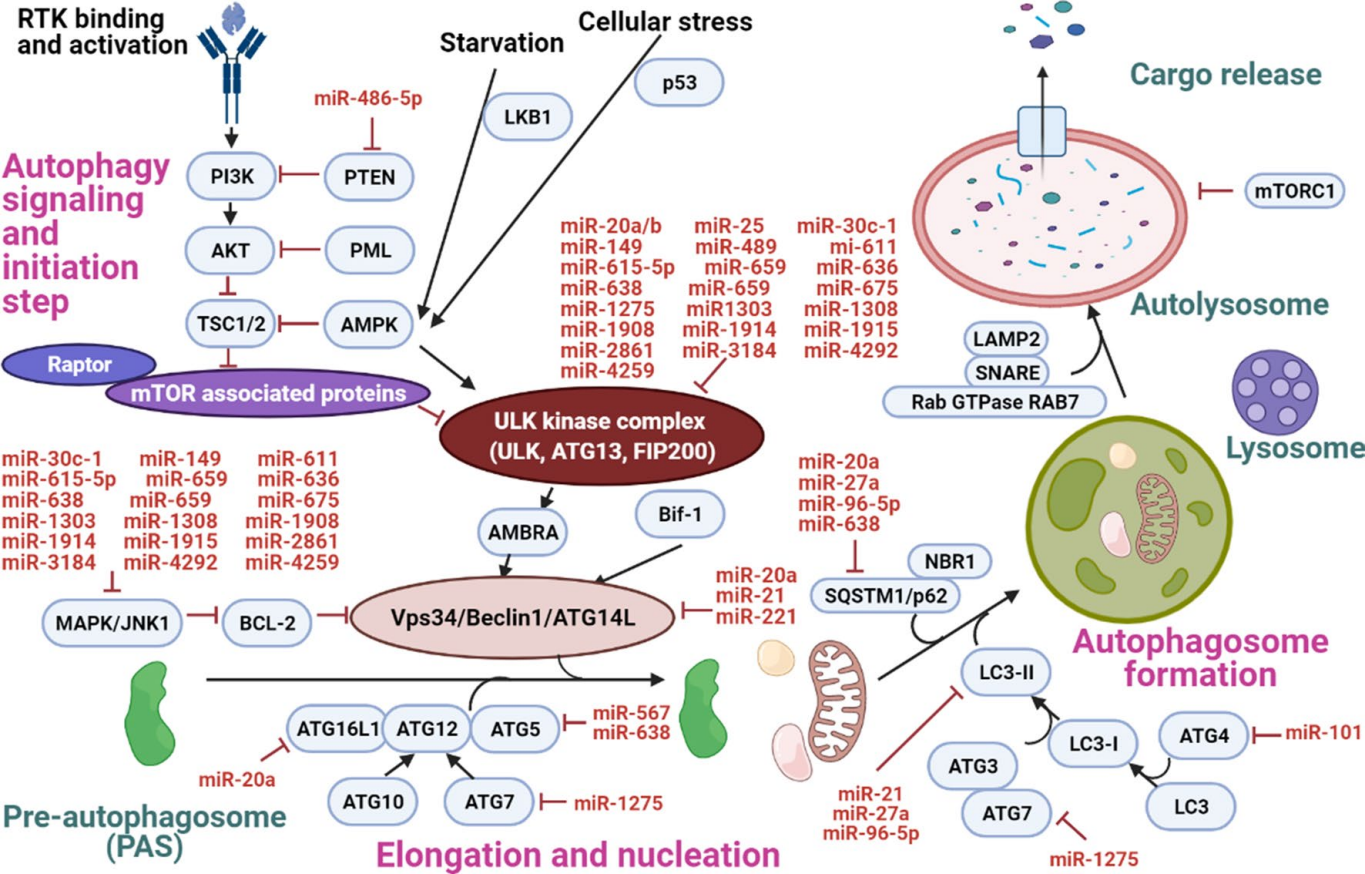
Roles of miRNAs in modulating the autophagy process in human breast cancer. MiR-486-5p is said to downregulate PTEN and this activates PI3K/Akt signaling pathway to inhibit autophagy activity [56]. An in vitro study reported eighteen miRNAs were involved in downregulating the signaling hub which covers ULK1 and these miRNA include miR-30c-1, miR-149, miR-611, miR-615-5p, miR-659, miR-636, miR-638, miR-659, miR-675, miR-1303, miR-1308, miR-1908, miR-1914, miR-1915, miR-2861, miR-3184, miR-4292 and miR-4259 [67]. This group of miRNAs was also found to downregulate the signalling hub which involves Beclin-1 by inhibiting the MAPK/JNK1 signalling pathway [67]. Another five miRNAs were reported to inhibit the ULK kinase complex and these include miR-20a and miR-20b [72], miR-25 [68], miR-489 [37] and miR-1275 [69]. On the other hand, Beclin-1 expression was found to be negatively correlated to the expression of miR-20a [74], miR-21 [75] and miR-221 [76]. MiR-20a could also suppress ATG16L1 expression [74] while miR-567 and miR-638 could negatively regulate ATG5 expression [78, 79]. Overexpression of miR-101 was linked to ATG4 downregulation [80] whereas upregulation of miR-1275 was related to ATG7 suppression [69], and both ATG4 and ATG7 are important in the LC3-II production [16]. Other miRNAs which were involved in suppressing cellular LC3-II production include miR-21 [75], miR-27a [82] and miR-96-5p [83] while miR-638 was found to be involved in promoting LC3-II production [81]. Four miRNAs were reported to be involved in suppressing SQSTM1/p62 expression and these miRNAs include miR-20a [74], miR-27a [82], miR-96-5p [83] and miR-638 [81]

In this review, a total of 41 miRNAs reported from 26 various in vitro, in vivo and clinical studies (Table 1) were included to discuss their roles in regulating autophagy process in human breast cancer development. In general, these autophagy-regulating miRNAs could modulate cellular autophagy (Fig. 3) by regulating (1) autophagy initiation, (2) elongation and nucleation steps moderated by autophagy-related proteins (ATGs), (3) autophagosome formation, and (4) expression of other proteins which do not involve directly in autophagy development. Out of these 41 reported miRNAs, 11 autophagy-regulating miRNAs were further reported to play vital roles in regulating treatment response in breast cancer (Table 2).

**Table 1:** Summary of miRNAs involved in regulating autophagy in human breast cancer (n = 41)

Roles of miRNAs in regulating autophagy and tumorigenesis	miRNAs	Downstream targets (Affected autophagy steps)	Study designs (Data source/cell lines/sample size, n)	Study summary	References
Promote both tumorigenesis and autophagy	miR-23a		In vitro secondary cell lines (MDA-MB-231, MDA-MB-453, T47D, SKBR3, BT549, MCF-10A and MCF-7); in vivo female Balb/c nude mice study (n = unclear for each group)	Overexpression of miR-23a downregulated (p < 0.05) XIAP expression and this promoted autophagy and breast cancer tumorigenesis in vitro and in vivo	[96]
	miR-23b-3p		In vitro secondary cell lines (MCF-7, ZR-75-1 and HCC1428); in vivo female Balb/c nude or NSG/NOD mice study (n = 4 for each group)	Downregulation of SLC6A14 by miR-23b-3p caused increased influx of acidic amino acids in the endocrine therapy resistant breast cancer cells and this promoted autophagy and tumorigenesis	[35]
	miR-126		Clinical sampling (breast cancer patients = 106); in vitro secondary cell lines (MCF-7 and MDA-MB-231); in vivo female Balb/c nude mice study (n = 9 for each group)	Overexpression of miR-126 downregulated IRS/Glut-4 signaling pathway and activated AMPK/autophagy pathway to promote tumorigenesis	[86]
	miR-638		In vitro secondary cell lines (KYSE450 ESCC and MCF-7); clinical sampling (breast cancer samples = 24, esophageal cancer tissues = 66)	Upregulation of miR-638 would significantly downregulate (p < 0.05) *DACT3* expression, which then promoted autophagy and tumorigenesis in both breast and esophageal cancers	[81]
Promote autophagy but suppress tumorigenesis	miR-125b-5p		In vitro secondary cell lines (MCF-7); in vivo female Balb/c nude mice study (n = 6 for each group)	MiR-125b-5p negatively regulated PAD2 (p < 0.05) and this sensitized the breast cancer cells to tamoxifen and docetaxel treatment by accelerating apoptosis and autophagy	[66]
Suppress autophagy but promote tumorigenesis	miR-20a		Online clinical data (breast cancer = 694, normal = 83); clinical sampling (n = 30 for cancer or normal tissues); in vitro secondary cell lines (MDA-MB-231 and MCF-7); in vivo female Balb/c nude mice study (n = 12 for each group)	miR-20a level was negatively correlated to *BECN1*, *ATG16L1* and *SQSTM1* expression level. Elevated miR-20a increased DNA mutation and tumorigenesis by decreasing autophagy activities	[74]
	miR-21		In vitro secondary cell line (MCF-7)	Knockdown of miR-21 would enhance autophagy and improve breast cancer cells sensitivity to tamoxifen and fulvestrant by inhibiting PI3K/AKT/mTOR pathway	[75]
	miR-25		In vitro secondary cell lines (MCF-7 and MCF-10A); in vivo female NOD/SCID mice (n = 6 for control or treated group)	Upregulation of miR-25 reduced autophagy by reducing *ULK1* expression (p < 0.05). This led to chemoresistance	[68]
Suppress autophagy but promote tumorigenesis	miR-96-5p		In vitro secondary cell lines (MDA-MB-231, MCF-7, BT-549, HS578T, T47D, ZR-75-1 and MCF-10A)	Overexpression of miR-96-5p significantly suppressed (p < 0.05) autophagy and apoptosis, and increased tumorigenesis	[83]
	miR-137		In vitro secondary cell lines (SKBR3, MCF-7 and MDA-MB-231)	Overexpression of miR-137 significantly (p < 0.05) apoptosis and autophagy and promoted cancer cells tumorigenesis	[99]
	miR-221		In vitro secondary cell lines (MDA-MB-231, MCF-7, T47D, ZR-751, SKBR-3 and HMEC); in vivo athymic female mice (n = 5 for each group)	Elevation of miR-221 downregulated beclin-1 (p < 0.05) in vitro and in vivo. This caused reduced autophagy but increased tumorigenesis and cancer aggressiveness	[76]
	miR-224-5p		Clinical sampling (metastatic breast cancer = 30, non-metastatic breast cancer = 35, normal control = 25); in vitro secondary cell lines (MDA-MB-231 and MCF-7)	Introduction of miR-224-5p suppressed autophagy by reducing Smad4 expression (p < 0.05). High miR-224-5p level was found in metastatic breast cancer patients than normal control or patients with non-metastatic lesions	[101]
	miR-486-5p		In vitro secondary cell lines (MCF-7 and MDA-MB-231)	Upregulation of miR-486-5p would downregulate PTEN expression (p < 0.05) and autophagy but enhanced AKT signaling pathway	[56]
Suppress autophagy but promote tumorigenesis	miR-638		Case–control (breast cancer or normal control, each had 47 samples); bioinformatics target prediction	Downregulation of miR-638 might be associated with good disease prognosis and slow disease prognosis by increasing autophagy activity	[79]
Suppress both autophagy and tumorigenesis	Let-7a	Un-reported	In vitro secondary cell line (MDA-MB-231)	Overexpression of Let-7a significantly (p < 0.05) increased apoptosis, reduced autophagy, and cell proliferation in vitro	[102]
	miR-20a miR-20b		Clinical sampling (breast cancer tissues and normal tissues = 19); in vitro secondary cell lines (MCF-7, MCF-10A and MDA-MB-231)	Overexpression of miR-20a and miR-20b suppressed (p < 0.05) autophagy and tumorigenesis	[72]
	miR-26b		Clinical sampling (breast cancer tissues and normal tissues = 3); in vitro secondary cell line (MCF-7)	Increased expression of miR-26b downregulated DRAM1 protein expression in breast cancer cell and this reduced autophagy and sensitized cancer cells to irradiation	[92]
	miR-27a		In vitro secondary cell lines (MDA-MB-231 and MCF-7)	Introduction of antagonist of miR-27a increased (p < 0.05) LC3-II and p62 expression in vitro, increased autophagy and chemoresistance	[82]
Suppress both autophagy and tumorigenesis	miR-101		In vitro secondary cell line (MCF-7)	Overexpression of miR-101 downregulated *STMN1*, *RAB5A* and *ATG4D*. This inhibited autophagy and promoted tamoxifen (4-OHT) induced cells apoptosis	[80]
	miR-107		Clinical sampling (breast cancer patients = 62); in vitro secondary cell lines (MDA-MB-231, MDA-MB-453, MCF-10A and MCF-7); in vivo female Balb/c nude mice study (n = 5 for each group)	In breast cancer tissues and cell lines, miR-107 was downregulated (p < 0.01) and this was associated with increased tumorigenicity. Overexpression of miR-107 downregulated HMGB1 in vitro and in vivo and inhibited autophagy	[36]
	miR-129-5p		In vitro secondary cell line (MCF-7)	Upregulation of miR-120-5p significantly suppressed (p < 0.05) HMGB1 expression. This led to autophagy downregulation and increased chemosensitivity against taxol	[61]
	miR-200c		Clinical sampling (breast cancer patients = 35); in vitro secondary cell lines (MDA-MB-231, BT549, BT474, MCF-10A and MCF-7)	Ectopic expression of miR-200c downregulated UBQLN1 and this blocked radiation-induced autophagy and sensitized cancer cells to radiotherapy	[93]
Suppress both autophagy and tumorigenesis	miR-489		In vitro secondary cell line (T47D, MCF-7, MDA-MB-231, MDA-MB-468, MDA-MB-361, Hs578T and ZR-75-1); in vivo athymic female mice (n = 5 for each group); clinical sampling (breast cancer patients = 14)	In vitro and in vivo findings suggested that upregulation of miR-489 downregulated *ULK1* and *LAPTM4B*, which then suppressed autophagy and increased cancer cells sensitivity towards doxorubicin	[37]
	miR-567		Clinical sampling (breast cancer tissues = 98); in vitro secondary cell lines (SKBR3 and BT474); in vivo male Balb/c nude mice study (n = 4 for each group)	Introduction of miR-567 downregulated ATG5 expression in vitro and in vivo, and this suppressed autophagy and sensitized cancer cells to trastuzumab	[78]
	miR-1275		Clinical sampling divided into 3 cohorts (breast cancer samples = 161, normal tissues = 127); in vitro secondary cell lines (MDA-MB-231 and MCF-7); in vivo Balb/c nude female mice (n = 6 for each group)	Overexpression of miR-1245 would downregulate *ATG7* and *ULK1 *in vitro and in vivo, which then suppressed autophagy and tumorigenesis. Downregulation of miR-1275 by circCDYL in breast cancer patients led to poor survival	[69]
Suppress both autophagy and tumorigenesis	miR-30c-1 miR-149 miR-611 miR-615-5p miR-659 miR-636 miR-638 miR-659 miR-675 miR-1303 miR-1308 miR-1908 miR-1914 miR-1915 miR-2861 miR-3184 miR-4292 miR-4259		In vitro secondary cell line (MDA-MB-231)	Introduction of BIK interference would result in the upregulation of a number of autophagy-regulating miRNAs which were shown to regulate several key autophagy-related proteins like ULK1/2, LC3-II and MAPK3	[67]

A total of 41 miRNAs from 26 published in vitro, in vivo and clinical studies were included in this review to discuss their roles in modulating autophagy in human breast cancer and to explore how does this influence the disease development and treatment response. 30 miRNAs were reported to suppress both autophagy and breast cancer tumerigenicity and these include let-7a [102], miR-20a and miR-20b [72], miR-26b [92], miR-27a [82], miR-101 [80], miR-107 [36], miR-129-5p [61], miR-200c [93], miR-489 [37], miR-567 [78], miR-1275 [69], and miR-30c-1, miR-149, miR-611, miR-615-5p, miR-659, miR-636, miR-638, miR-659, miR-675, miR-1303, miR-1308, miR-1908, miR-1914, miR-1915, miR-2861, miR-3184, miR-4292 and miR-4259 [67]. Four miRNAs which were shown to enhance both autophagy and breast cancer progression include miR-23a [96], miR-23b-3p [35], miR-126 [86] and miR-638 [81]. Nine miRNAs were found to suppress autophagy but enhance breast cancer development and these miRNAs include miR-20a [74], miR-21 [75], miR-25 [68], miR-96-5p [83], miR-137 [99], miR-221 [76], miR-224-5p [101], miR-486-5p [56] and miR-638 [79]. One miRNA was shown to promote autophagy and suppress breast cancer tumerigenicity and this miRNA is miR-125b-5p [66]. Out of all these miRNAs, miR-20a was found to suppress both autophagy and tumorigenesis in a study [72] but in another study [74], miR-20a was said to promote cancer progression despite it suppressed cellular autophagy. Another miRNA which was shown to have different overall effects on autophagy regulation in breast cancer is miR-638, in which it was demonstrated to accelerate both autophagy and tumorigenesis in a study [81] but in another case–control study [79], miR-638 was shown to inhibit autophagy and promote breast cancer progression

Upregulation;  Downregulation

**Table 2:** Roles of miRNAs in influencing treatment response in breast cancer by regulating autophagy activities (n = 11)

Treatment types	Influence on treatment response	miRNA(s)	Autophagy activities ( )	Proposed mechanisms/evidences	References
Chemotherapy	Resistant-promoting	miR-25		miR-25 downregulated ULK1 to suppress autophagy and promote epirubicin resistance in breast cancer cells	[68]
	Sensitivity-promoting	miR-27a		miR-27a suppressed LC3-II and p62 expressions to inhibit autophagy and sensitized cancer cells to doxorubicin and paclitaxel	[82]
		miR-129-5p		miR-129-5p suppressed HMGB1 to inhibit autophagy and sensitized breast cancer cells to taxol treatment	[61]
		miR-489		miR-489 downregulated ULK1 to block autophagy and sensitized breast cancer cells to doxorubicin treatment	[37]
Endocrine therapy	Resistant-promoting	miR-23b-3p		miR-23b-3p downregulated SLC6A14 to promote autophagy and tamoxifen resistance	[75]
		miR-21		miR-21 could promote PI3K/AKT/mTOR pathway to inhibit autophagy and promote resistance towards both tamoxifen and fulvestrant	[35]
	Sensitivity-promoting	miR-101		miR-101 suppressed ATG4D, STMN1 and RAB5A expressions to block autophagy and promote tamoxifen-induced cells death	[80]
Endocrine and chemotherapy	Sensitivity-promoting	miR-125b-5p		miR-125b-5p suppressed PAD2 expression to promote both autophagy and apoptosis. This sensitized cancer cells to both tamoxifen and docetaxel	[66]
Radiotherapy	Sensitivity-promoting	miR-26b		miR-26b downregulated DRAM1 expression to inhibit autophagy and sensitize breast cancer cells to irradiation	[92]
		miR-200c		miR-200 downregulated UBQLN1 to block autophagy and sensitized breast cancer cells to irradiation	[93]
Targeted therapy	Sensitivity-promoting	miR-567		miR-567 suppressed autophagy by downregulating ATG5 to sensitize cancer cells to trastuzumab treatment	[78]

11 miRNAs were reported to play essential roles in regulating treatment responses in breast cancer by modulating the cellular autophagy activities [35, 37, 93, 61, 66, 68, 75, 78, 80, 82, 92]. The treatment responses which could be modulated by these autophagy-regulating miRNAs include responses towards chemotherapy [37, 61, 66, 68, 82], endocrine therapy [35, 66, 75, 80], radiotherapy [92, 93] and targeted therapy [78]. Out of these 11 miRNAs, only two miRNAs, namely, miR-23b-3p [75] and miR-125b-5p [66], were shown to upregulate autophagy activities while the rest were demonstrated to inhibit autophagy [35, 37, 61, 68, 78, 80, 82, 92, 93].  Upregulation;  Downregulation

### Roles of miRNAs in regulating autophagy initiation

Activation of PI3K/Akt/mTOR signaling pathway has been proven to play an essential role in downregulating cellular autophagy activity by activating mTOR protein that suppresses ULK kinase complex activity (Fig. 2) [55]. An in vitro study reported in year 2018 showed that upregulation of miR-486-5p would downregulate PTEN and this resulted in the activation of the PI3K/Akt/mTOR signaling pathway to suppress autophagy and enhance tumorigenesis [56]. PTEN is responsible in dephosphorylating PIP3, an intermediate lipid signaling molecule in the PIP3/Akt/mTOR signaling pathway, and this causes the cessation of signals along this cellular pathway [55].

ULK kinase complex comprises ULK1, ATG13, ATG101 and FIP200 proteins and it is responsible in phosphorylating the downstream Beclin-1 protein to initiate the autophagy activity [49]. In an in vitro study [67] involving triple-negative human breast cancer cell line, MDA-MB-231, the authors reported that 18 miRNAs could play a role in downregulating the signaling hub involving ULK1 and these miRNAs include miR-30c-1, miR-149, miR-611, miR-615-5p, miR-659, miR-636, miR-638, miR-659, miR-675, miR-1303, miR-1308, miR-1908, miR-1914, miR-1915, miR-2861, miR-3184, miR-4292 and miR-4259. Three other miRNAs which were reported to be able to downregulate ULK1 include miR-25 [68], miR-489 [37] and miR-1275 [69]. By suppressing ULK1 expression, these 21 miRNAs could potentially block autophagy induction by reducing the activation of the AMBRA protein, in which AMBRA is important in phosphorylating the downstream Beclin-1 containing class III PI3K [16]. For miR-489, other than being shown to negatively regulate autophagy activity, it was also demonstrated to reduce breast cancer resistance towards doxorubicin in vitro and in vivo [37]. In most of the mentioned studies [37, 67, 69], it was shown that autophagy blockage by the corresponding miRNAs would lead to decreased breast cancer tumorigenesis. However, in the combined in vivo and in vitro study that involved miR-25 [68], it was shown that the introduction of miR-25 mimics would restore cellular proliferation and increase cancer cells resistance towards epirubicin, apart from abrogating autophagy. The opposite effects of autophagy on the breast cancer tumorigenesis suggested that autophagy could play the role of a double-edged sword in promoting cancer progression or suppression [70]. FIP200 is another important component of the ULK kinase complex [71], and this protein was found to be negatively regulated by miR-20a and miR-20b [72]. As a result, suppression of FIP200 by these two miRNAs led to autophagy suppression by blocking autophagy initiation step and this also inhibited cancer progression in vitro [72].

Beclin-1 protein is a key component of the class III PI3K complex which plays an essential role in the autophagy initiation step [16]. Bcl-2 is an anti-apoptotic protein that could bind to Beclin-1 to inactivate it and phosphorylation of Bcl-2 by MAPK/JNK1 would dissociate it from Beclin-1, allowing Beclin-1 to initiate the autophagy signalling activity [73]. Same like the case of ULK1 which has been discussed previously, the in vitro study by Ruiz Esparza-Garrido et al. reported that about 18 miRNAs were found to be able to suppress the signaling hub involving MAPK/JNL1 [67], and thus, this would allow the dephosphorylated Bcl-2 to bind to Beclin-1 to inactivate its activity [73]. Besides, three other miRNAs were shown to negatively regulate Beclin-1 and this include miR-20a [74], miR-21 [75] and miR-221 [76]. Compared to the previous single study that reported 18 potential miRNAs which would suppress Beclin-1 activity, autophagy and tumorigenicity, these three studies reported enhanced tumerigenicity despite causing autophagy inhibition [74–76]. This suggested that degree of autophagy might influence the survival of cancer cells and excessive autophagy might in fact reduce tumerigenicity [77]. Another possible reason to explain the observance of increase tumerigenicity despite autophagy suppression could be found in one of the studies [75], in which the study discovered that apart from suppressing Beclin-1, miR-21 would also downregulate PTEN to enhance PI3K/Akt signaling pathway. Increase activity of the PI3K/Akt pathway eventually increased tumerigenicity and caused increase resistance of the breast cancer cells towards tamoxifen and fulvestrant in vitro [75]. Therefore, the overall effect on whether an autophagy-regulating miRNA would increase or decrease tumerigenicity also depends on the downstream target or signaling cascade in which it could have exerting effect.

### Roles of miRNAs in modulating elongation and nucleation steps during autophagy development by regulating the expression of autophagy-related proteins (ATGs)

Another group of key proteins which play vital roles in modulating autophagy process is the autophagy-related proteins (ATGs) [49]. A number of miRNAs have been reported to target various ATGs (Fig. 3) and these include miR-20a (ATG16L1) [74], miR-567 and miR-638 (ATG5) [78, 79], miR-101 (ATG4) [80] and miR-1275 (ATG7) [69]. ATG16L1 has been proven to be able to bind to WIPI2 protein directly and this helps recruiting the ATG12/ATG15/ATG16L1 protein complex to the pre-autophagosome (PAS) [46]. MiR-21a was found to downregulate ATG16L1 expression and this suppressed autophagy and increased breast cancer carcinogenesis [74]. ATG5, on the other hand, could be negatively regulated by miR-567 [78] and miR-638 [79], and this resulted in autophagy inhibition. In the study which reported the autophagy inhibitory role of miR-567, it was shown that the breast cancer cells showed increased sensitivity towards trastuzumab treatment in vitro following upregulation of miR-567 and thus, this miRNA is said to suppress both autophagy and breast cancer tumerigenicity [78]. However, in the latter case-control study that involved miR-638, it was shown that ATG5 could be negatively regulated by miR-638, but, low miR-638 expression was associated with poor disease prognosis and enhanced disease progression [79]. This suggested that autophagy suppression by miR-638 was linked to increased cancer tumerigenicity [79], and a more detailed mechanistic study is needed to validate this case–control study finding. ATG4 is a cysteine protein which is involved in converting LC3 to LC3-I before LC3-II can be produced [39]. Overexpression of miR-101 would downregulate ATG4 expression, and, this would lead to autophagy and tumerigenicity suppression [80]. In addition, increased expression of miR-101 would also increase the sensitivity of breast cancer cell line, MCF-7, towards tamoxifen treatment, evidenced by increased cells death [80]. Another miRNA that was shown to involve in regulating ATG expression is miR-1275 in which this miRNA has been demonstrated to suppress ATG7 expression, and this resulted in both autophagy and tumerigenicity suppression [69]. ATG7 has been shown to be involved in converting LC3-I to LC3-II by facilitating the covalent binding of LC3-I with phosphatidylethanolamine at the PAS membrane [39]. Therefore, the suppression of ATG7 would affect the LC3-II production and this would affect the formation of mature autophagosome [49].

### Roles of miRNAs in regulating mature autophagosome formation

On the other hand, several miRNAs were reported to directly regulate the cellular level of LC3-II and miR-638 was shown to directly increase the expression of LC3-II in vitro to increase cellular autophagy and tumorigenesis [81]. This study finding contradicts another study finding which stated that miR-638 was involved in downregulating autophagy by targeting ATG5 and ATG-2B [79]. In another study [67], upregulation of miR-638 was found to enhance cellular LC3-II level, but, the authors described that this phenomenon could be related to other stimulus induction as most of the autophagy-related transcripts were downregulated when miR-638 expression was increased. Therefore, more functional assay may need to be conducted to clarify the exact role of miR-638 in regulating cellular autophagy and tumorigenesis. For miRNAs that were shown to correlate to decreased cellular LC3-II level, three miRNAs were reported and these include miR-21 [75], miR-27a [82] and miR-96-5p [83]. For miR-21, apart from being demonstrated to decrease cellular LC3-II level, miR-21 was also shown to suppress Beclin-1 expression and these combined effects would inhibit autophagy in vitro [75]. The upregulation of both miR-27a and miR-96-5p were proven to reduce cellular level of LC3-II to suppress autophagy and tumorigenesis [82, 83]. In addition, overexpression of miR-27a was also shown to improve the breast cancer cells sensitivity towards doxorubicin and paclitaxel in vitro [82]. Besides, both miR-27a and miR-96-5p were proven to reduce the cellular level of SQSTM1 protein in vitro [82, 83]. SQSTM1 is an important ubiquitination protein that tags the macromolecules or cargo to be degraded so that these substances can be recognized and bind to the autophagy initiating complex [18]. Downregulation of SQSTM1 would impair substrates delivery to the autophagosome and this would results in the accumulation of aggregated protein and thus, this would eventually lead to inflammatory and degenerative disease [18]. Other miRNA which was shown to suppress cellular SQSTM1 includes miR-20a and this miRNA also downregulates other autophagy related proteins like ATG16L1 and Beclin 1 [74].

Even though multiple miRNAs have been reported to play essential roles in regulating autophagy initiation, elongation, nucleation and autophagosome formation, however, to the best of our knowledge, there is a lack of study which has reported any miRNA that could target the key proteins (LAMP2, SNARE, Rab GTPase RAB7) which are important in regulating the fusion of autophagosome and lysosome to form autolysosome.

### Roles of miRNAs in regulating autophagy by modulating expressions of other proteins that do not involve directly in autophagy development

Apart from regulating the key proteins involved in the autophagy signaling pathway (Fig. 3), there are also some miRNAs which could modulate the autophagy activities by regulating other cellular proteins which are not involved directly in the autophay signaling pathway. SLC6A14 is a type of basic amino acid transporter that is negatively regulated by miR-23b-3p and the downregulation of this transporter would result in the increased influx of acidic amino acids via another transporter, SLC1A2 [35]. The disruption in the cellular amino acid level may promote autophagy [84] and SLC6A14 blockage has been shown to promote autophagy in colon cancer [85]. As a result, miR-23b-3p was shown to enhance both autophagy and tumorigenesis, and increase resistance of the breast cancer cells towards hormonal drugs like fulvestrant and tamoxifen [35]. MiR-126 was reported to downregulate the IRS/Glut-4 signaling pathway and this would cause cellular energy deprivation [86]. It has been widely established that glucose depletion is an essential factor that triggers autophagy [87] and thus, miR-126 is said to induce autophagy by activating the AMPK/autophagy signaling cascade [86]. PAD2 is a corepressor of tumour suppressor p53 protein [88] and p53 can negatively regulate PI3K/Akt signaling pathway [89]. MiR-125b-5p was reported to downregulate PAD2 and this would increase p53 expression that inhibits PI3K/Akt signaling [66]. Blockage of PI3K/Akt signaling pathway would enhance autophagy as mTOR is downregulated and the net effect was increased autophagy, decreased cancer cells proliferation and increased cancer cells sensitivity towards tamoxifen and docetaxel [66]. HMGB1 is an autophagy-regulating protein that functions to displace Bcl-2 to bind to Beclin-1, in order to activate Beclin-1 for autophagy induction [90]. An in vitro study involving MCF-7 cancer cell line showed that overexpression of miR-129-5p would downregulate HMGB1 and therefore, this suppressed both autophagy and tumorigenesis [61]. In addition, upregulation of miR-129-5p was also found to improve MCF-7 sensitivity towards taxol in vitro [61]. Another miRNA which was reported to downregulate HMGB1 is miR-107 [36] and like miR-129-5p, miR-107 suppressed both autophagy and cancer cells proliferation by downregulating HMGB1 expression.

DRAM1 is identified as a direct downstream target of p53 and its detailed role in autophagy modulation is still not fully understood [91]. DRAM1 may be involved in increasing lysosomal acidification or inhibiting Akt signaling pathway to promote cellular autophagy [91]. In a combined clinical and in vitro study [92], it was shown that overexpression of miR-26b would negatively regulate DRAM1 expression and this resulted in decreased autophagy, tumorigenesis and improved cancer cells sensitivity towards radiotherapy. In another combined clinical and in vitro study [93], ectopic expression of miR-200c was shown to downregulate UBQLN1 to suppress autophagy and tumorigenesis, and sensitized the breast cancer cells towards radiotherapy. UBQLN proteins like UBQLN1 and UBQLN4 have been reported to play some roles during the formation of autophagosome and these proteins might also help in the fusion of autophagosome and lysosome to form autolysosome [94]. XIAP is an anti-apoptotic protein in which its role in autophagy modulation remains controversy [95]. MiR-23a was reported to downregulate XIAP to increase autophagy and tumorigenesis in a published study finding [96], however, the exact molecular mechanism on how XIAP dowregulation led to enhanced autophagy was not explored further. One possible explanation is that XIAP might play certain role in inhibiting MDM2/p53 signaling pathway to block autophagy induction [95] and by suppressing XIAP expression, autophagy could be induced [96].

Fundc1 is an important mammalian mitochondrion membrane protein which plays an essential role in recruiting LC3 to the mitochondrion to initiate mitophagy [97]. It has been reported that Fundc1 could also upregulate the expression of autophagy-related proteins like Beclin-1, ATG5 and ATG7, and apoptotic-related proteins like BAX [98]. In view of the multiple autophagy roles that could be played by Fundc1 protein, a group of researchers investigated the potential role of miR-137 in modulating Fundc1 expression and they found that this protein could be negatively regulated by miR-137 [99]. Downregulation of Fundc1 following miR-137 overexpression would subsequently promote tumorigenesis [99] and the reason of increased tumorigenesis could be related to the downregulation of the apoptotic-related genes following Fundc1 downregulation [98]. Another protein which has been reported to be involved in regulating the transcription of autophagy-related genes is Smad4 and it is part of the TGF-β regulated signaling pathway [100]. The activation of the TGF-β/Smad4 signaling pathway will increase the expression of several key autophagy-related proteins like ATG5, ATG6 and ATG7 [100]. MiR-224-5p was reported to suppress Smad4 expression and autophagy in vitro and this resulted in increased cancer cells tumerigenicity [101]. Like Fundc1, Smad4 also involves in upregulating expression of apoptotic-related proteins like Bcl, BIK and BIM [100] and thus, suppression of Smad4 would lead to apoptosis inhibition and cause increase in the cancer progression [101]. Let-7a, on the other hand, was reported to downregulate both autophagy and cancer cells tumerigenicity in vitro but its direct downstream target was unreported [102]. However, let-7a has been reported to promote autophagy in other solid cancers like gastric cancer [26] and lung cancer [29]. Therefore, more study is needed to confirm whether let-7a would suppress autophagy in human breast cancer cells.

### Roles of autophagy‐regulating miRNAs in modulating treatment response in breast cancer

From all the previously discussed autophagy-regulating miRNAs, 11 miRNAs were further reported to be involved in regulating treatment responses towards chemotherapy [37, 61, 66, 68, 82], endocrine therapy [35, 66, 75, 80], radiotherapy [92, 93] and targeted therapy [78] in breast cancer (Table 2). Four miRNAs, namely, miR-25, miR-27a, miR-129-5p and miR-489 were reported to suppress autophagy in breast cancer cells [37, 61, 68, 82]. However, miR-25 was the only miRNA which was demonstrated to promote chemoresistant in breast cancer [68] while the other three miRNAs were proven to promote chemosensitivity in breast cancer [37, 61, 82]. As autophagy has been reported to be able to exert both tumour-promoting and tumour-suppressing effects [61], it is therefore not surprising that autophagy induction or inhibition would promote treatment resistance in some study while in other study, autophagy dysregulation could promote sensitivity towards a specific cancer therapy. Similar phenomenon was observed when it was shown that both miR-21 and miR-101 would suppress cellular autophagy activities but the former miRNA would promote resistance towards endocrine therapy [35] while the latter one would promote sensitivity towards endocrine therapy [80]. Compared to miR-21 and miR-101, miR-23b-3p was the only miRNA which was shown to promote both autophagy and resistance towards endocrine therapy in breast cancer [75]. Other than miRNA, other non-coding RNAs like long non-coding RNA H19 has also been reported to be able to promote both autophagy and tamoxifen resistance in breast cancer [103]. On the other hand, miR-125b-5p was recognized as the autophagy-promoting miRNA that could induce sensitivity towards both chemotherapy and hormonal therapy by upregulating cellular autophagy and apoptosis [66]. This suggests that miR-125b-5p could be possibly further studied to investigate its potential to be used in clinical trial to improve sensitivity of the breast cancer patients towards both chemotherapy and endocrine therapy.

For radiotherapy, miR-26b and miR-200c were reported to be autophagy-inhibiting miRNAs that promote radiosensitivity [92, 93]. These findings probably suggested that autophagy could be important in providing nutrients for the cancer cells to maintain cellular homeostasis during radiotherapy [63]. Similarly, miR-567 was also shown to suppress autophagy and promote sensitivity towards targeted therapy in breast cancer [78]. Again, this further supported the essential role of autophagy in ensuring the cancer cells survival when targeted therapy is administered [64]. One important point to take note of is that it can be observed that autophagy induction or inhibition in chemotherapy and endocrine therapy could either promote treatment resistance or treatment sensitivity but in both radiotherapy and targeted therapy, autophagy inhibition seems to promote treatment sensitivity only than treatment resistance.

## Autophagy-regulating miRNAs as potential breast cancer biomarkers and therapeutic agents: applications, challenges and recommendations

As discussed in the previous section, it can be clearly seen that a number of miRNAs (Table 1) has been demonstrated to influence the breast cancer progression or treatment response by modulating the cellular autophagy process. By understanding the relationships between a specific miRNA and its effect on the cellular autophagy modulation and tumorigenesis, it helps enabling this miRNA to be employed as the breast cancer biomarker [37, 79]. Take miR-125b-5p as an example, upregulation of this miRNA would suppress breast cancer tumorigenesis and sensitize the breast cancer cells towards tamoxifen and docetaxel by accelerating both apoptosis and autophagy [66]. The autophagy enhancement was achieved by downregulating PAD2 protein expression [66] and this protein is a corepressor for tumour suppressor protein p53 [88]. Therefore, by monitoring the level of miR-125b-5p in the human breast cancer patients, it may help to monitor the disease progression and predict the response towards hormonal and chemotherapy. Besides, miRNA can also be utilized as a novel therapeutic agent to tackle breast cancer progression [35, 99]. For instance, miR-107 has been proven to inhibit breast cancer cells proliferation, migration and autophagy in vivo and in vitro by targeting HMGB1 [36]. This generates a possibly to increase the expression of miR-107 level in the human breast cancer patients to slow breast cancer progression [36]. In short, the use of miRNAs as cancer biomarkers and therapeutic agents may help allow disease detection and progress monitoring, which will then help the patients to improve their treatment response and survival [104].

Autophagy can be a double-edged sword in either promoting cancer progression or suppression [61, 70]. Autophagy may help generating nutrients for the cancer cells to grow and remove dysfunctional cellular macromolecules but excessive autophagy may lead to cellular death [55, 77]. Even though numerous studies have proposed the potential roles of autophagy-regulating miRNAs as cancer biomarkers or therapeutic agents, one problem faced is that some autophagy-regulating miRNA seems to exert different effects on the breast cancer tumorigenesis in different studies. By taking miR-20a as an example, in the study reported by Liu et al., it was shown that miR-20a would suppress autophagy but enhance breast cancer progression in vivo and in vitro [74]. However, in another study, it was demonstrated that miR-20a would suppress both autophagy and breast cancer progression [72]. Both studies reported autophagy suppression by miR-20a but this miRNA would exert different effects on the breast cancer tumorigenesis in two different studies [72, 74]. Therefore, it is inconclusive to say whether miR-20a upregulation in the breast cancer patient is a good or bad sign and thus, more further study is needed to validate the exact role of miR-20a clinically, before this miRNA can be used as a breast cancer biomarker or therapeutic agent.

Another problem with the potential use of miRNAs as cancer biomarker is that it requires many detailed and independent testings, before a panel of effective and specific cancer biomarkers can be introduced [105]. To the best of our knowledge, currently there is no a panel of autophagy-regulating miRNAs which has gone through multiple testings to prove its effectiveness and specificity. So, it is suggested that future study can focus on the list of autophagy-regulating miRNAs which have been reported to play roles in human breast cancer development, and from the list, more detailed study can be conducted to evaluate the suitability of these miRNAs as breast cancer biomarkers. As for the use of miRNA as potential cancer therapeutic agent, several problems like suitable delivery methods and unwanted off-target effects are still remain unsolved [106], and thus, there is still a long way to go before autophagy-regulating miRNAs can be certified safe to be employed as the novel breast cancer therapeutic agent.

## Conclusions

This review effectively summarizes the findings from various in vitro, in vivo and clinical studies on the roles of a number of autophagy-regulating miRNAs in influencing the human breast cancer progression and treatment response. By modulating the cellular autophay process, these miRNAs could actually suppress or enhance the breast cancer progression. Therefore, these miRNAs have great potentials to be developed into useful breast cancer biomarkers or new therapeutic agent. To make this happens, more detailed mechanistic and clinical trials should be conducted to evaluate the safety, specificity, sensitivity and effectiveness of these miRNAs as breast cancer biomarkers and therapeutic agents.

## Data Availability

All data was included in the manuscript.

## Abbreviations

AMBRA: Autophagy and beclin-1 regulator
AMPK: Adenosine monophosphate-activated protein kinase
ATG16L1: Autophagy related 16 like 1
ATG4D: Autophagy related 4D cysteine peptidase
BAX: Bcl-2-associated X protein
Bcl-2: B cell lymphoma 2
BECN1: Beclin-1
Bif-1: Endophilin
BIK: Bcl-2 interacting killer protein
BIM: Bcl-2 like protein 11
CMA: Chaperon-mediated autophagy
DACT3: Dishevelled binding antagonist of beta catenin 3
DFCP1: Zinc-finger FYVE domain-containing protein 1
DRAM1: DNA damage-regulated autophagy modulator protein 1
ER: Endoplasmic reticulum
ESCRT/III: Endosomal sorting complexes required for transport I and III
FIP200: FAK family kinase-interacting protein of 200 kDa
FOXO1: Forkhead box protein O1
Fundc1: FUN14 domain-containing protein 1
FYCO1: FYVE and coiled-coil domain-containing protein 1
GFAP: Glial fibrillary acidic protein
Glut-4: Glucose transporter type 4
HGS: Hepatocyte growth factor-regulated tyrosine kinase substrate
HMGB1: High mobility group box 1 protein
HOPS: Homotypic fusion and protein sorting protein complex
HSC70: Heat shock cognate 71 kDa protein
HSP90: Heat shock protein 90
IRS: Insulin receptor substrate
JNK1: c-Jun N-terminal kinase 1
LAMP-A2: Lysosome-associated membrane protein type 2a
LAPTM4B: Lysosomal protein transmembrane 4 beta
LC3-I/II: LC3-phosphatidylethanolamine conjugate I/II
LKB1: Serine/threonine-protein kinase STK11
LYNUS: Lysosome nutrient sensing machinery
MAPK: Mitogen activated protein kinase
MDM2: Mouse double minute 2 homolog
MiRNA: MicroRNA
mRNA: Messenger RNA
mTOR: Mammalian target of rapamycin
NBR1: Next to BRCA1 gene 1 protein
ncRNA: Non-coding RNA
NBR1: Next to BRCA1 gene 1 protein
NOD: Non-obese diabetic
PAD2: Peptidylarginine deiminase 2
PAS: Pre-autophagosome
PE: Phosphatidylethanolamine
PI3K: Phosphoinositide 3-kinase
PIP2: Phosphatidylinositol-4,5-bisphosphate
PIP3: Phosphatidylinositol-3,4,5-bisphosphate
PKB/Akt: Protein kinase B
PLEKHM1/2: Pleckstrin homology domain-containing family M member 1/2
PML: Promyelocytic leukemia protein
PTEN: Phosphatase and tensin homolog
RAB5A: Ras-related protein Rab-5A
RBCC1: RB1-inducible coiled-coil protein 1
RILP: Rab-interacting lysosomal protein
RNA: Ribonucleic acid
RTK: Receptor tyrosine kinase
SCID: Severe combined immunodeficiency disease
SLC6A14: Sodium- and chloride-dependent neutral and basic amino acid transporter B
Smad4: SMAD family member 4
SNARE: Soluble NSF attachment protein receptor
SNCA: α-Synuclein
SQSTM1: Sequestosome-1
STMN1: Stathmin 1
TECPR1: Tectonin beta-propeller repeat containing 1
TFEB: Transcription factor EB
TGF-β: Transforming growth factor β
TSC1/2: Tuberous sclerosis 1/2
UBQLN1: Ubiquilin-1
ULK1: Unc-51 like autophagy activating kinase
VPS15/34: Vacuolar protein sorting 15/34
WIPI2: WD repeat domain phosphoinositide-interacting protein 2
XIAP: X-linked inhibitor of apoptosis protein
3′-UTR: 3′-Untranslated region
4-OHT: 4-hydroxytamoxifen
